# Metabolic response of Insulinoma 1E cells to glucose stimulation studied by fluorescence lifetime imaging

**DOI:** 10.1096/fba.2020-00014

**Published:** 2020-06-19

**Authors:** Gianmarco Ferri, Marta Tesi, Federico Massarelli, Lorella Marselli, Piero Marchetti, Francesco Cardarelli

**Affiliations:** ^1^ Laboratorio NEST ‐ Scuola Normale Superiore Pisa Italy; ^2^ Department of Clinical and Experimental Medicine Islet Cell Laboratory University of Pisa Pisa Italy; ^3^ Dipartimento di Fisica "E. Fermi" Università di Pisa Pisa Italy

**Keywords:** glucotoxicity, hyperglycemia, INS‐1 E, insulin secretion, metabolic imaging, NADH

## Abstract

A cascade of highly regulated biochemical processes connects glucose stimulation to insulin secretion in specialized cells of mammalian pancreas, the β‐cells. Given the importance of this process for systemic glucose homeostasis, noninvasive and fast strategies capable to monitor the response to glucose in living cells are highly desirable. Here, we use the phasor‐based approach to Fluorescence Lifetime IMaging (FLIM) microscopy to quantify the ratio between protein‐bound and free Nicotinamide adenine dinucleotide (phosphate) species in their reduced form (NAD(P)H), and the Insulinoma cell line INS‐1E as a β‐like cellular model. Phasor‐FLIM analysis shows that the bound/free ratio of NAD(P)H species increases upon pulsed glucose stimulation. Such response is impaired by 48‐hours preincubation of cells under hyperglycemic conditions. Phasor‐FLIM concomitantly monitors the appearance of long‐lifetime species (LLS) as characteristic products of hyperglycemia‐induced oxidative stress.

**Abbreviations**
ATP/ADP: Adenine TriPhospate/Adenine DiPhosphate
ELISA: Enzyme‐Linked Immunosorbent Assay
ETC: Electron Transport Chain
FLIM: Fluorescence Lifetime Imaging
GSIS: Glucose Stimulated Insulin Secretion
HEK 293: Human Embryonic Kidney 293 cells
INS‐1E: Insulinoma cell line
LLS: Long Lifetime Species
mGPDH: mitochondrial Glycerol‐3‐Phosphate DeHydrogenase
mMDH: mitochondrial Malate DeHydrogenase
NAD(P)H: Nicotinamide Adenine Dinucleotide (Phosphate), reduced
ROS: Reactive Oxygen Species, MCT1, MonoCarboxylate Transporter 1
RPMI: Roswell Park Memorial Institute

## 1 INTRODUCTION

The biochemical process by which β‐cells orchestrate Glucose‐Stimulated Insulin Secretion (hereafter referred to as GSIS) is pivotal to the maintenance of glucose systemic homeostasis.^1^ In brief, after being transported into the cell cytoplasm, glucose gets readily phosphorylated and metabolically digested during glycolysis; the products of glucose oxidation, such as pyruvate and Nicotinamide Adenine Dinucleotide in its reduced form (ie NADH), are shuttled together with other substrates into mitochondria by the mitochondrial glycerol phosphate dehydrogenase (mGPDH) and the mitochondrial malate dehydrogenase (mMDH) transporters^2^ to participate to the Krebs’ cycle and produce large quantities of NADH.^3^ NADH then acts as a potent electron carrier (being oxidized into NAD+) during the mitochondrial oxidative phosphorylation, fueling the production of adenosine triphosphate (ATP) molecules. Increased ATP/ADP ratio levels induce closure of plasma‐membrane‐associated ATP‐sensitive potassium channels, which in turn induces depolarization of the plasma membrane and activation of voltage‐sensitive calcium channels. Ca^2+^ influx finally promotes and sustain insulin secretion by mobilization of insulin secretory granules from the cytoplasm to the fusion with the plasma membrane.^4^

It is possible to assess quantitatively the efficiency of the overall process described above by monitoring in vitro the amount (and timing) of the secreted insulin with respect to the amount of administered glucose. These assays provide a useful experimental platform to monitor the general responsiveness of β‐cells to glucose and its possible alteration in the pathological condition^5^ although they average out the biochemical processes that occur in between stimulation and secretion. On the other hand, the idea to monitor directly the β‐cell biochemistry during GSIS by optical microscopy of suitably labelled molecules (eg by introducing genetically‐encoded fluorescent proteins) is challenged by the need not to alter the chemical identity and endogenous stoichiometry of the biochemical components of the whole metabolic process.^6^, ^7^, ^8^ In this context, the phasor approach to Fluorescence Lifetime IMaging (FLIM) microscopy can be a useful tool as it exploits the intrinsic signal of NAD(P)H molecules.^9^ In fact, endogenous NAD(P)H molecules can be selectively excited by using a 2‐photon light source tuned in the 700‐740‐nm range, with minimal perturbation/damage of the sample. In addition, while intensity‐based measurements may contain artifacts due to the heterogeneity of fluorophore concentration and to differing quantum yields of NAD(P)H species,^10^, ^11^ the measured lifetime is minimally affected by cell absorption/scattering and/or fluctuation in excitation intensity and can selectively discriminate the free and protein‐bound forms of NAD(P)H molecules.^12^ In turn, then, the ratio of bound/free NAD(P)H species can be used to define the overall metabolic state of cells (eg low or high bound/free ratio) and its possible alteration during processes such as disease progression, differentiation, cell fate, cell division.^13^, ^14^, ^15^, ^16^ Specifically in the context of insulin secretion, the phasor‐FLIM analysis on NAD(P)H species was used to monitor the metabolic status of intact human and/or mouse Langerhans islets under different stimuli.^17^, ^18^, ^19^ In particular, in a recent pioneering work by Gregg and collaborators,^17^ phasor‐FLIM analysis revealed an increase in the ratio of bound/free NAD(P)H species in both human and mouse islets in response to glucose stimulation, an effect then impaired by aging. The observed shift toward NAD(P)H in its ‘bound’ form in the intact islet is generally attributed to the response of the β‐cell to the glucose stimulation, although it is not currently possible to distinguish between different endocrine cell types within such a complex system. Notwithstanding the undoubted importance of using intact islets, immortalized β‐like cellular models have become a useful tool to get insights into the behavior of β‐cells,^20^, ^21^ provided that the selected model recapitulates properly the main features of actual β‐cells in response to different experimental conditions (eg glucose stimulation). In this regard, here we test Insulinoma 1E (INS‐1E) β‐like cells and their metabolic response in terms of bound/free NAD(P)H upon glucose stimulation by phasor‐FLIM analysis. Our experiments show that the bound/free ratio of NAD(P)H species in INS‐1E cells increases upon pulsed glucose stimulation, recapitulating what was observed in the intact islet (refs. 17). As expected based on insulin‐secretion assays (that we also performed as control), chronic preincubation of cells to hyperglycemic conditions (30 mmol/L glucose for 48 hours) significantly impairs the characteristic INS‐1E metabolic response to glucose pulsed stimulation. Finally, phasor‐FLIM analysis is able to monitor, within the same experiment, the appearance of characteristic long‐lifetime species (LLS) (metabolic products of oxidative stress,^22^, ^23^) in cells chronically exposed to hyperglycemic conditions.

## 2 MATERIALS AND METHODS

### 2.1 Cell culture

INS‐1 E cells^20^ (kindly provided by Prof. C. Wollheim, University of Geneva, Medical Center) between passage 80 and 90 and A549 cells (passage 15‐25) were maintained in culture at 37°C, 5% CO_2_ in RPMI 1640 medium containing 11.1 mmol/L D‐glucose, 10 mmol/L HEPES, 2 mmol/L L‐Glutamine, 100 U/mL penicillin‐streptomycin, 1 mmol/L sodium‐pyruvate, 50 µmol/L tissue culture grade β‐mercaptoethanol (all purchased from Gibco, ThermoFisher). 24 hours before label‐free imaging, cells were plated onto sterilized and fluorescence‐microscopy‐suitable dishes (IbiTreat µ‐Dish 35‐mm, Ibidi). For hyperglycemic condition, INS‐1E cells were grown in complete RPMI medium with 30 mmol/L glucose for 48 hours. Glucose stimulation for phasor‐FLIM experiments were performed incubating INS‐1E for 45 minutes in Krebs’ solution at 2.5 mmol/L glucose prior to microscope acquisitions, then switched to Krebs’ 16.7 mmol/L glucose and followed for 45 minutes.

### 2.2 Insulin secretion assay

Insulin release was evaluated as previously described in Ref. 24. In brief, INS‐1E cells were preincubated in Krebs’ solution containing 2.5 mmol/L glucose for 30 minutes, then cells were exposed to the same medium at 2.5 or 16.7 mmol/L glucose for 45 minutes. At the end, the supernatant was collected and stored at −20°C until insulin measurement. Insulin levels were quantified by the High Range Rat Insulin ELISA kit following the manufacturer's instructions (Mercodia AB). Data are represented as ng of released insulin normalized by the amount of cells.

### 2.3 Two‐photon microscopy and phasor‐FLIM measurements

Phasor‐FLIM measurements were carried out with the ISS Flimbox system embedded in an Olympus FluoView 1000‐ASW‐2.0 confocal laser microscope coupled with a two‐photon Ti:sapphire laser with 80‐MHz repetition rate (Chameleon Vision, Coherent). A 690 BS was used to separate excitation and emission from label free sample. NADH was excited at 710 nm and the emission was collected by using a 60× planApo water immersion objective (NA = 1.2) in the 400 − 500 nm range. Calibration of the ISS Flimbox system was performed by measuring the known mono‐exponential lifetime decay of Fluorescein at pH 11.1 (ie 4 ns upon excitation at 710 nm, collection range: 500‐600 nm). A stock of 100 mmol/L Fluorescein solution in EtOH was prepared and diluted in NaOH at pH 11.1 for each calibration measurement. For each measurement FLIM data are collected until about 100 counts are acquired on the average in the 256 × 256‐pixels image. The final acquisition time was typically in the order of 1‐2 minutes, depending on the signal intensity from the sample.

### 2.4 Data analysis

The theory behind phasor FLIM has been described previously in Ref. 13. In brief, each pixel in phasor plots is defined by coordinates *g* and *s*, calculated from the fluorescence intensity decay of each pixel of the FLIM image by using the transformations defined in Ref. 25, considering the first harmonic (ie 80 MHz) of the laser repetition rate. Every possible lifetime measured in samples is mapped into the phasor plot. The semicircle depicted around phasor plot is called universal circle which represents all of the possible single exponential lifetimes which may be calculated. For a multiexponential lifetime, within the distribution plot of the phasor the lifetimes appear as a linear combination of the expected single exponential lifetimes (NAD(P)H in the ‘free’ and ‘bound’ forms, in this case), making the plot lie inside the universal circle. All possible weighting of the two molecular species give phasors distributed along a straight line joining the phasors of the two species. In the case of three molecular species, all the possible combinations are contained in a triangle where the vertices correspond to the phasor of the pure species. For two and three component analysis of fractional NAD(P)H distribution and LLS identification we followed detailed steps reported in Ref. 25. All phasor transformation and the data analysis of FLIM data are performed using SimFCS v. 4 software developed at the LFD (Laboratory for Fluorescence Dynamics).

## 3 RESULTS

### 3.1 Preliminary fluorescence intensity analysis of NAD(P)H species in INS‐1E cells

**FIGURE 1:**
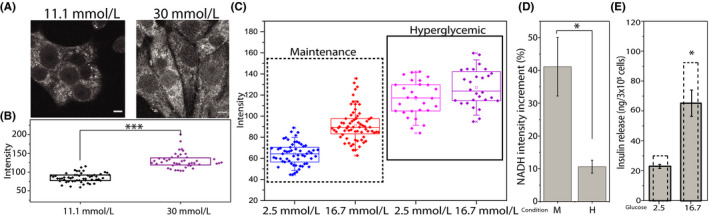
Autofluorescence intensity and insulin release analysis. A, Representative images of NADH auto‐fluorescence intensity in INS‐1E in maintenance condition (11.1 mmol/L glucose) and in INS‐1E cultured for 48 h in hyperglycemic condition (30 mmol/L glucose). Scale bar: 5 μm. B, Box plots for different culturing conditions. Each point represents the total NADH intensity of a single cell. Boxes represent 25 and 75 percentiles, lines represent median, whiskers represent SD (***P < .001, Mann‐Whitney test). C, Box plots for different culturing conditions. Each point represents the total NADH intensity of a single cell. Boxes represent 25 and 75 percentiles, lines represent median, whiskers represent SD. D, NADH intensity percentage increment in maintenance (M) and hyperglycemic (H) conditions, calculated as fold increment in NADH intensity measured between 2.5 and 16.7 mmol/L; *P < .05. E, Insulin release measured by ELISA‐kit assay in low (2.5 mmol/L) and high (16.7 mmol/L) glucose concentration in cells pretreated for 48 h with 30 mmol/L glucose. Stimulation was carried out following protocol described in Section 2. Data are the means ± SE of three separate measurements. Dashed columns represent measured insulin release in maintenance condition. *P < .05 compared with high glucose (16.7 mmol/L) of maintenance condition

By using a two‐photon excitation source tuned at 710 nm flashed on living cultured (and unlabeled) cells, the main contribution to the total detected auto‐fluorescence signal arises from NAD(P)H species, which are optically active molecules with most of the fluorescence emission in the 400‐500 nm range.^26^ Under typical maintenance culturing conditions (ie complete RPMI medium supplemented with 11.1 mmol/L glucose), an intrinsic fluorescence signal from INS‐1E cells is clearly detectable (Figure 1A). It derives mainly from cytoplasmic regions, which appear with a peculiar patterned staining (presumably belonging to mitochondria). Cell nuclei, instead, can be distinguished as ellipsoidal regions with a sensibly lower auto‐fluorescence signal (Figure 1A). Overall, the intracellular distribution of detected signals reflects the expected localization of NAD(P)H species and well agrees with previous measurements on different cellular systems.^10^, ^27^ The same cells cultured for 48 hours in hyperglycemic conditions (30 mmol/L glucose), which are known to impair β‐cells metabolism and physiology,^27^, ^28^ show a similar autofluorescence pattern, but with absolute intensity values that appear significantly increased compared to maintenance conditions (130.2 ± 19.9, 84.7 ± 12.2 respectively, *P* < .0001) (Figure 1B). This finding is in line with previous observations correlating glucose concentration in the medium with intracellular autofluorescence signal.^10^, ^27^ As described in detail in Section 2 and according to previous reports,^24^ a protocol for acute glucose stimulation of INS‐1E was used. The total autofluorescence intensity of INS‐1E cells (Figure 1C) increases significantly upon exposure to 16.7 mmol/L glucose concentration with respect to 2.5 mmol/L glucose after both the above‐mentioned culturing conditions (48 hours at 11.1 or 30 mmol/L glucose). However, this increment is significantly higher in cells previously kept at 11.1 mmol/L glucose, compared to those cultured at 30 mmol/L glucose (respectively, 41.6 ± 10.6% and 8.9 ± 2.0%, *P* = .007, Figure 1D). Accordingly, acute glucose‐stimulated insulin release in response to 16.7 mmol/L glucose was significantly higher in INS‐1E cells cultured for 48 hours at 11.1 mmol/L glucose with respect to those kept at 30 mmol/L glucose (Figure 1E), indicating that in this latter case cells experienced “glucose toxicity”.^29^

### 3.2 Validation of phasor‐FLIM analysis of NAD(P)H species in INS‐1E cells in response to glucose stimulation

**FIGURE 2:**
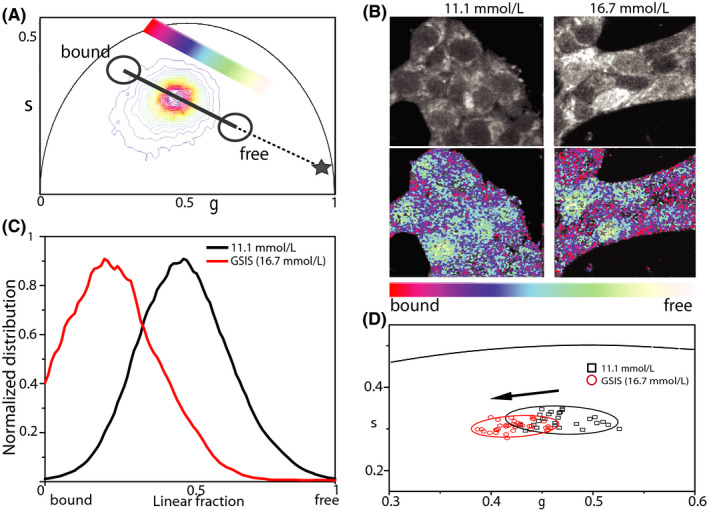
Phasor FLIM analysis. A, Phasor plot of control and stimulated cells. Color‐bar defines the metabolic path from NAD(P)H in the bound state (red/magenta) to NAD(P)H in the free state (green/white). Grey symbol represents the position in the phasor plot of pure NADH in solution. B, Exemplary images of total NAD(P)H intensity of INS‐1E cell clusters (top line) in 11.1 mmol/L glucose and GSIS. On bottom line, same images colored in accordance to the color bar defined below. C, Distributions of the free and bound NAD(P)H species for all pixels of acquired image in B for 11.1 mmol/L and GSIS condition. D, Scatter plot of the average values of distinct phasor distributions, each relative to distinct acquired cells. Black squares represent cells in maintenance condition, in red circles stimulated cells. Standard deviation is depicted as 90% confidential ellipsoid in accordance to aforementioned colors

**FIGURE 3:**
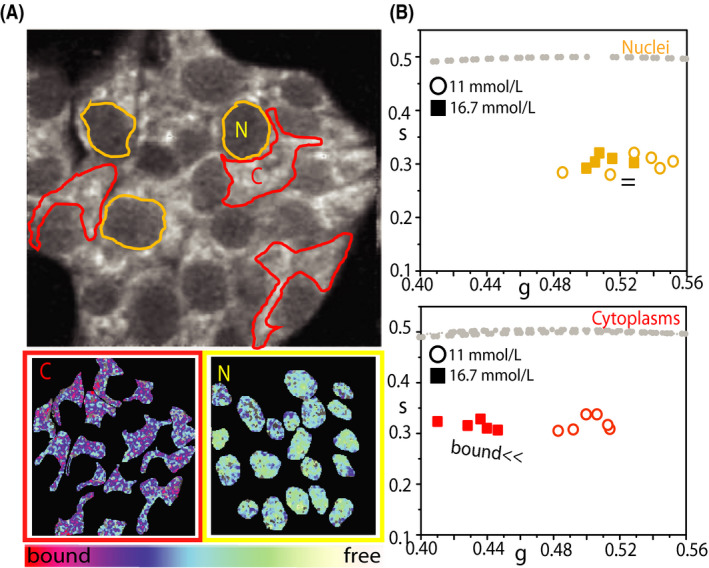
Segmentation analysis on INS‐1E cell upon glucose stimulation. A, Top: Exemplary NAD(P)H intensity image of a cluster of INS‐1E cells. Examples of ROI for segmentation analysis are reported: nuclei are identified as yellow ROI (N) while cytoplasmic regions are identified as red ROI (C). Nuclei are selected based on their ellipsoidal shape and typical lower NAD(P)H intensity signal. Bottom: Example of segmentation analysis performed on the cell cluster above, with color‐mapped NAD(P)H FLIM‐phasor reported. Color‐bar defines the metabolic path from NAD(P)H in the ‘bound’ state (red/magenta) to NAD(P)H in the ‘free’ state (green/white). B, Scatter plot of the average values of distinct phasor distributions obtained by segmentation analysis for nuclei (yellow) and cytoplasmic regions (red) for cell cultured in maintenance condition (circle, n = 6 acquisitions) and stimulated with 16.7 mmol/L glucose (solid square, n = 5 acquisitions), each relative to distinct analyzed cell clusters

As already stated above, intensity‐based measurements of NAD(P)H species inevitably contain artifacts due to the heterogeneity of fluorophore concentration and to differing quantum yields of NADH in the free and bound (to proteins) form.^30^ FLIM, instead, reports on the fluorophore's microenvironment and, in this case, can discriminate between ‘free’ and ‘protein‐bound’ NAD(P)H species within the cell. Phasor FLIM analysis applied to INS‐1E cells under maintenance culturing conditions yields a reference position of the NAD(P)H bound/free lifetime ratio (Figure 2A) (see Figure S1 for the phasor‐plot position of NADH molecules dissolved in aqueous solution). Visual inspection of FLIM maps suggests the expected prevalence of NAD(P)H molecules in the ‘bound’ form within the cytoplasm while nuclei are enriched NAD(P)H molecules in the ‘free’ form (Figure 2B, left panels). This readout is used to test cell response to glucose stimulation following the protocol described above. More in detail, once exposed at 16.7 mmol/L glucose, cells were imaged for approximately 45 minutes. As showed in Figure 2B‐C, glucose stimulation produces a clear shift of the NAD(P)H bound/free lifetime ratio toward higher values. In particular, in Figure 2D we report a comparative analysis of the phasor coordinates of unstimulated (black squares, n = 28) and stimulated cells (red circles, n = 35), represented by single data points, in which variations along both ‘*g*’ and ‘*s*’ axes of the phasor plot can be appreciated. At this point, by exploiting the imaging potential of 2‐photon FLIM, we performed spatial segmentation analysis of data to extract the specific contribution of the cytoplasm (and nucleus) of cells to the observed metabolic shift (Figure 3A). The obtained scatter plots of the average values of phasor distributions from the nucleus (yellow, top graph) and cytoplasm (red, bottom graph) are reported in Figure 3B: it is clear that the cytoplasm of cells is the location where the metabolic shift toward an higher bound/free NAD(P)H lifetime ratio does take place (compare empty and full data points in Figure 3B, bottom panel).

**FIGURE 4:**
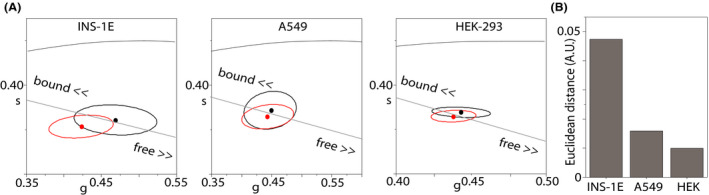
Phasor coordinates shift in different cell lines. A, Phasor FLIM plot for different cell lines (INS‐1E, A549, HEK‐293). In each phasor plot confidential ellipsoids are depicted in representation of distinct measures of unstimulated (black) and stimulated (red) cells. Ellipsoid central points (average value) are reported. B, Euclidean distance calculated as geometrical distance between ellipsoid central points

The metabolic response upon glucose stimulation observed in insulin‐secreting INS‐1E cells was compared to similar measurements performed in nonsecreting cells, namely A549 (lung tumor‐related cells) and HEK 293 cells (human embryonic kidney cells) (Figure 4). Both A549 and HEK293 cells exposed to the same treatments (ie pulsed stimulation with 16.7 mmol/L glucose to mimic secretion) did not show any significant increase in the bound/free NAD(P)H lifetime ratio of NAD(P)H (complete analysis for A549 is reported in Figure S2) calculated as the Euclidean distance between the ellipsoid central points of unstimulated and stimulated cells (average values, Figure 3B). To exclude possible effects induced by the simple switch from RPMI to Krebs’ buffer independently from medium glucose concentration, we cultured INS‐1E in Krebs at 11.1 mmol/L glucose concentration as control, obtaining similar results (Figure S3).

### 3.3 Reduced response to glucose stimulation under hyperglycemic conditions

**FIGURE 5:**
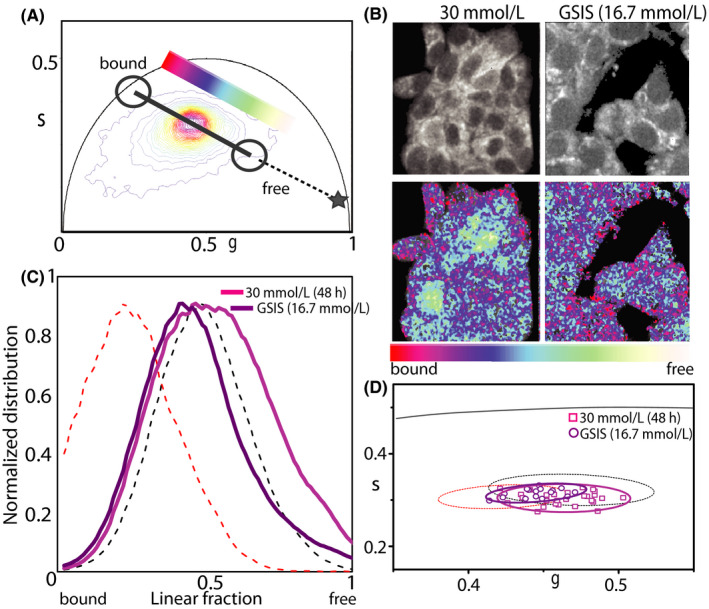
Phasor FLIM analysis on hyperglycemic culturing condition. A, Exemplary phasor plot of cells cultured in hyperglycemic condition and stimulated. Colorbar defines the metabolic path from NAD(P)H in the ‘bound’ state (red/magenta) to NAD(P)H in the ‘free’ state (green/white). B, Exemplary images of total NAD(P)H intensity of cells cluster (top line) after 48 h in 30 mmol/L glucose and after GSIS. On bottom line, same images colored in accordance to the color bar defined below. C, Distributions of the free and bound NAD(P)H species for all pixels of acquired image in B for 30 mmol/L and GSIS condition. D, Scatter plot of the average values of distinct phasor distributions, each relative to distinct acquired cells cluster. In magenta squares n = 30 cells in 48 h hyperglycemic culturing condition, in violet circles n = 20 stimulated cells. Standard deviation is depicted as 90% confidential ellipsoid in accordance to aforementioned colors. Confidential ellipsoid relative to standard culturing condition (dotted black line) and induced stimulation starting from standard condition (dotted red line) are also depicted

**FIGURE 6:**
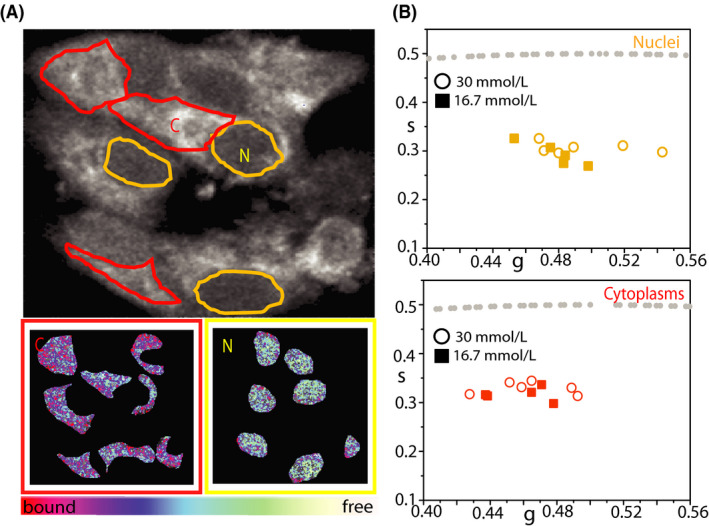
Segmentation analysis on INS‐1E cells preincubated in hyperglycemic conditions. A, Top: Exemplary NAD(P)H intensity image of a cluster of INS‐1E cells cultured in 30 mmol/L glucose for 48 h. Examples of ROI for segmentation analysis are reported: nuclei are identified as yellow ROI (N) while cytoplasmic regions are identified as red ROI (C). Nuclei are selected based on their ellipsoidal shape and typical lower NAD(P)H intensity signal. Bottom: Example of segmentation analysis performed on the cell cluster above, with color‐mapped NAD(P)H FLIM‐phasor reported. Color‐bar defines the metabolic path from NAD(P)H in the ‘bound’ state (red/magenta) to NAD(P)H in the ‘free’ state (green/white). B, Scatter plot of the average values of distinct phasor distributions obtained by segmentation analysis for nuclei (yellow) and cytoplasmic regions (red) in cells cultured in hyperglycemic condition (circle, n = 6 acquisitions) and stimulated with 16.7 mmol/L glucose (solid square, n = 5 acquisitions), each relative to distinct analyzed cell clusters

Based on what learned so far, we addressed INS‐1E response to glucose pulsed stimulation after exposing cells to hyperglycemic conditions (30 mmol/L glucose for 48 hours, see Section 2 for more details). In this experiment, cells show a sensibly reduced metabolic response in terms of phasor shift toward higher bound/free NAD(P)H lifetime ratios (Figure 5A). This can be visualized qualitatively by means of the color‐coded maps of intracellular NAD(P)H bound/free ratio (exemplary case in Figure 5B) and, quantitatively, by the corresponding bound/free ratio value for each measured cell(Figure 5C) and cumulative results from the whole population of observed cells (Figure 5D). Again, spatial segmentation analysis of data is useful to extract the specific contribution of the cytoplasmic and nuclear regions of cells (Figure 6A). The obtained scatter plots of the average values of phasor distributions from the nucleus (yellow, top graph) and cytoplasm (red, bottom graph) are reported in Figure 6B. As somewhat expected, the contribution to the metabolic response of the cytoplasmic regions (that was dominant upon glucose stimulation from maintenance conditions) is now almost completely impaired (compare empty and full data points in Figure 6B, bottom panel).

### 3.4 Reduced response to glucose stimulation under hyperglycemic conditions correlates with intracellular oxidative stress

**FIGURE 7:**
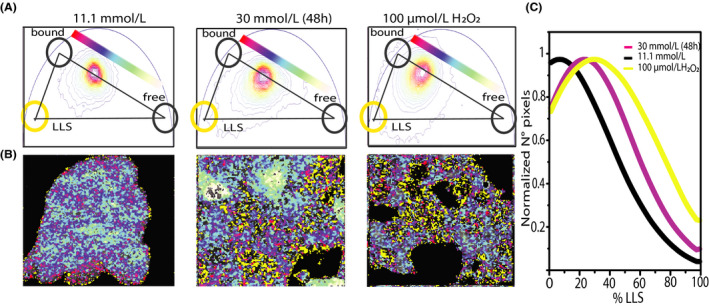
LSS species as oxidative stress signature induced by hyperglycemia. A, Three components analysis on phasor plot of cells cultured in 11.1 mmol/L glucose, exposed to high glucose medium for 48 h and treated with 100 μm H_2_O_2_ to stimulate ROS production. B, Exemplary images and relative lifetime‐based color map for NADH metabolic trajectories. Pixels with LLS species are colored in yellow, according to the position of yellow cursor in phasor plot. C, Resultant graph from three components analysis, in which percentage of LLS species is plotted vs the number of pixels in which long lifetime species are present for the three conditions

The phasor‐FLIM analysis gives access to additional metabolic information in the case that reactive oxygen species (ROS)‐induced damages are produced intracellularly, especially on lipids. Datta et al,^23^ in fact, identified the products of lipid oxidation by ROS as auto‐fluorescent, endogenous markers of stress, with a characteristic long fluorescent lifetime (ie approximately 8 ns). The presence of long lifetime species (LLS) generates a third auto‐fluorescent component along with those belonging to ‘free’ and ‘bound’ NAD(P)H but still with similar excitation and emission properties. Such additional contribution to the average measured lifetime induces a detectable alteration of the lifetime distribution in the phasor plot. More in detail, phasor plots in Figure 7A show the emergence of an elongation of the lifetime distribution toward the LLS‐specific position in the phasor plot in cells exposed for 48 hours to 30 mmol/L glucose with respect to those maintained at 11.1 mmol/L glucose. Phasor points which show the shift toward LLS were highlighted using a yellow cursor while the metabolic path connecting the characteristic ‘bound’ and ‘free’ states was shown using same color scheme represented in previous figures (Figure 7B; more details about the segmentation analysis used can be found in Figure S4). To confirm that the identified LLSs are a product of oxidative stress, cells cultivated under standard conditions were treated with 100 μmol/L of hydrogen peroxide. Massive ROS treatment with H_2_O_2_ induces a more pronounced shift toward LLS vertex respect to control and even respect to cell growth in 30 mmol/L, as confirmed by three component analysis reported in Figure 7C. The data show the fractional intensity contribution of the LLS for each pixels of the acquired images, that is larger in the 30 mmol/L condition and for 100 μmol/L H_2_O_2_ treatment respect to standard cultivation condition.

## 4 DISCUSSION

Phasor‐FLIM analysis of NAD(P)H species is used here as a label‐free optical approach to study the metabolic status of insulin‐secreting INS‐1E cells under maintenance culturing conditions and pulsed glucose stimulation (this latter applied starting from both maintenance and hyperglycemic conditions). Differently from NAD(P)H intensity analysis, FLIM has the potential to retrieve quantitative information about the ratio of NAD(P)H molecules in the ‘bound’ (eg to enzymes) and ‘free’ form, although with a few selected limitations, namely: (a) NADH and NAD(P)H species cannot be distinguished; (b) the metabolic response is reported in terms of bound/free ratio but changes can occur in both the numerator and denominator; (c) the approach, as used here, does not have native single‐enzyme or single‐pathway resolution (eg to study the respiratory chain with NAD(P)H‐FLIM, specific respiratory‐chain inhibitors need to be applied).

In this work, we show that INS‐1E cells respond to standard glucose stimulation by increasing the bound/free ratio of their intracellular pool of NAD(P)H molecules. This result recapitulates what observed by others in the entire human and mouse Langerhans islet^17^ and corroborates the idea that what observed in the islet well describes the contribution of β‐cells. Along this reasoning, INS‐1E response is consistent with a large amount of biochemical and genomic/proteomic data collected on β‐like and actual β‐cells. It is known, for instance, that finely tuned metabolic pathways are active in pancreatic β‐cells to funnel glucose carbons from glycolysis to the mitochondria for oxidative phosphorylation.^31^ Unlike most of the cells, β‐cells, in fact, lack the lactate transporter MCT1 and show reduced expression levels of lactate dehydrogenase, thus sensibly reducing the amount of glucose that is metabolized to lactate.^32^, ^33^ In addition, the mGPDH and mDH hydrogen shuttles that transport NADH from the cytoplasm to the mitochondria are particularly active in the β‐cell,^34^, ^35^ favoring NADH consumption by the Electron Transport Chain (ETC). Such a potentiated orientation toward oxidative phosphorylation metabolism is, indeed, a key biochemical signature of the β‐cell. It is thus not surprising that, in our experimental setup, nonsecreting cells show substantially no metabolic shift upon glucose stimulation.

Phasor‐FLIM is used here also to show that INS‐1E chronically exposed to hyperglycemia have a very reduced shift toward oxidative phosphorylation metabolism after glucose stimulation (data supported by a measured reduced GSIS with ELISA assay). In this regard, Haythorne and collaborators, by means of a multi‐omics functional approach, recently showed that mitochondrial metabolism is impaired in rodent islets and INS‐1 cells cultivated in hyperglycemic conditions.^27^ In addition, the hyperglycemic condition is known to induce the cellular production of reactive oxygen species (ROS) and the ROS‐related oxidative stress, especially by mitochondria.^36^ When β‐cells are exposed to hyperglycemic conditions for a prolonged time, the glucose metabolism is unbalanced and there is an increase of ROS derived from mitochondrial ETC.^29^, ^37^, ^38^, ^39^ Phasor‐FLIM analysis here successfully identifies the emergence of ROS‐induced damage in the context of INS‐1E dysfunction, paving the way to rapid, label‐free, and simultaneous measurements of cell metabolic status and ROS‐induced damage in β‐cells as well as in entire islets.

## AUTHOR CONTRIBUTIONS

GF performed research, analyzed data, wrote the manuscript; MT performed insulin secretion assays, analyzed data; FM cultivated the cells; LM, analyzed data, wrote the manuscript; PM designed research, analyzed data, wrote the manuscript; FC designed research, supervised research, analyzed data, wrote the manuscript.

## Supporting information

Fig S1‐S4
