# Supplementary material for: Metabolic response of Insulinoma 1E cells to glucose stimulation studied by fluorescence lifetime imaging
Source: FASEB Bioadv. 2020 Jun 19;2(7):409–18. doi: 10.1096/fba.2020-00014 (PMC7354695; doi:10.1096/fba.2020-00014)
Supplement: Supplementary file 1 — Fig S1‐S4 [file FBA2-2-409-s001.pdf]

Metabolic response of Insulinoma 1E cells to glucose stimulation studied by fluorescence lifetime imaging

Authors:

Gianmarco Ferri[a], Marta Tesi[b], Federico Massarelli[c], Lorella Marselli[b], Piero Marchetti[b] and Francesco Cardarelli [a,*]

Affiliations:

[a] Laboratorio NEST - Scuola Normale Superiore, Piazza San Silvestro 12, Pisa, Italy.

[b] Department of Clinical and Experimental Medicine, Islet Cell Laboratory, University of Pisa, Pisa, Italy.

[c] Department of Physics, University of Pisa, Pisa, Italy

[*]To whom correspondence should be addressed: francesco.cardarelli@sns.it (phone: +39 050 509698)

Supplementary information

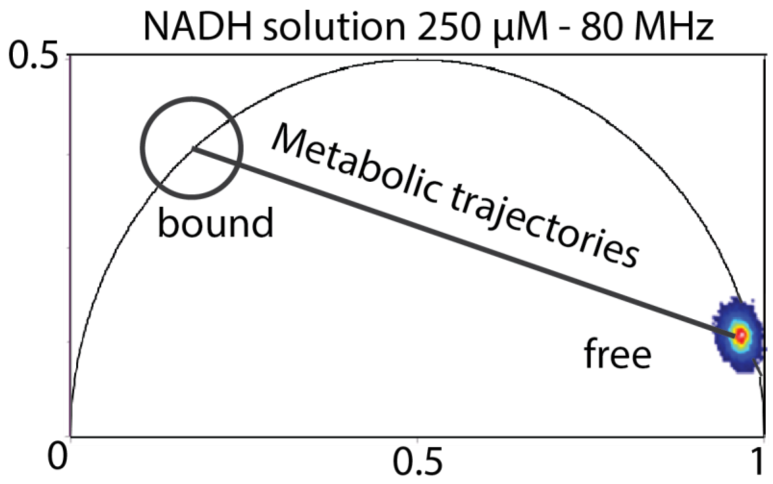

Suppl. Fig. 1 Phasor FLIM analysis on a solution of NADH. FLIM-phasor measurement on a 250 μM NADH free solution, used as reference for metabolic trajectories position.

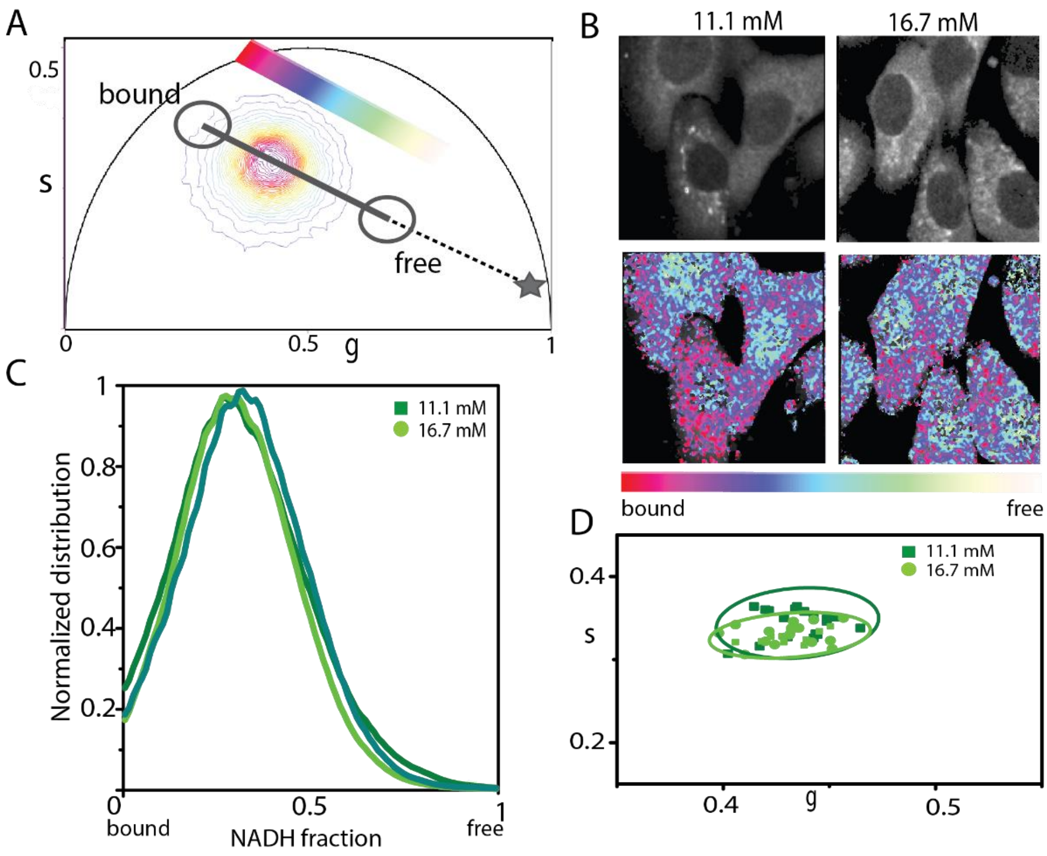

Suppl. Fig. 2 Phasor FLIM analysis of A549 cells. A) Total phasor plot of acquired cells, in maintenance condition and stimulated. Colobar defines the metabolic trajectories from NAD(P)H bound state (red/magenta) to NADH free state (green/white). B) Exemplary images of total NAD(P)H intensity of A549 cells (top line) in 11.1 mM, 2.5 mM and 16.7 mM glucose. On bottom line, same images colored in accordance to the color bar defined below. C) Scatter plot of the mean values of distinct phasor distributions, each relative to distinct acquired cells. Standard deviation is depicted as confidential ellipsoid according to aforementioned colors. D) Average distributions of the two NAD(P)H species for all pixels in each acquired image for 11.1 mM and stimulation.

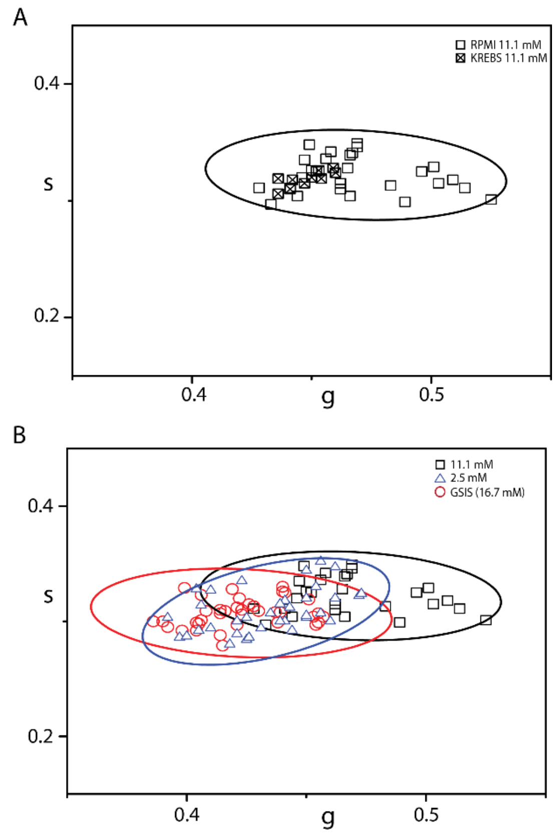

Suppl. Fig. 3 A) Scatter plot of the mean values of distinct phasor distributions, each relative to distinct acquired cells cluster. Black empty squares represent INS-1E in normal RPMI medium, while crossed squares represent INS-1E bathed in 11.1 mM Krebs' buffer for 1h. B) Scatter plot of distinct phasor represented as single points for 11.1 mM, 2.5 mM and stimulated 16.7 mM culturing conditions. Standard deviation is depicted as confidential ellipsoid according to colors in legend.

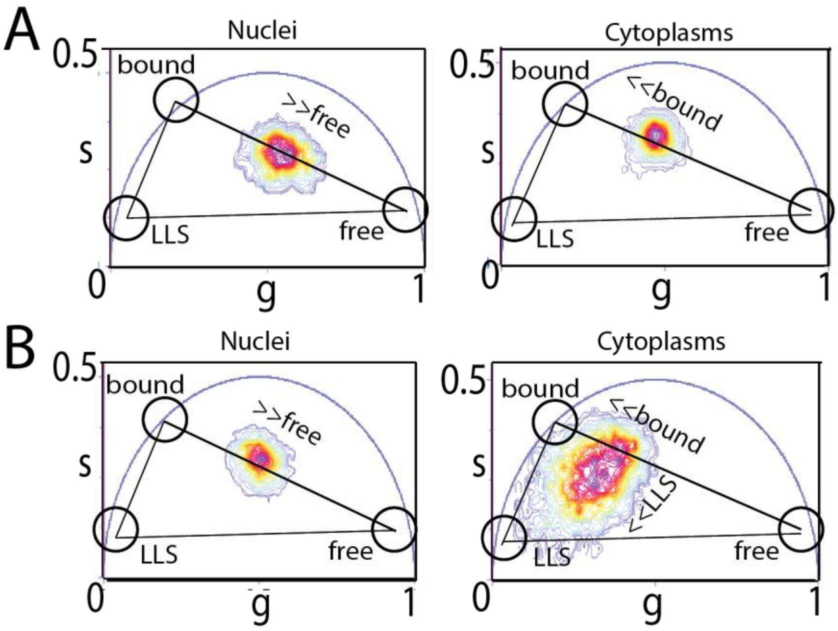

Suppl. Fig. 4 Segmentation analysis for LLS localization. A) Phasor plots resulted from segmentation analysis on 11mM cultured cells (as reported in Fig. 6 and 3) for nuclei and cytoplasms. B) Phasor plots resulted from segmentation analysis on 30 mM cultured cells (48h hyperglycemia) for nuclei and cytoplasms.
